# Diamond Molecular Balance: Ultra-Wide Range Nanomechanical Mass Spectrometry from MDa to TDa

**DOI:** 10.1021/acs.nanolett.5c02032

**Published:** 2025-06-19

**Authors:** Donggeun Lee, Seung-Woo Jeon, Chang-Hwan Yi, Yanghee Kim, Yeeun Choi, Sang-Hun Lee, Jinwoong Cha, Seung-Bo Shim, Junho Suh, Il-Young Kim, Dongyeon Daniel Kang, Hojoong Jung, Cherlhyun Jeong, Jae-pyoung Ahn, Hee Chul Park, Sang-Wook Han, Chulki Kim

**Affiliations:** † Center for Quantum Technology, 58975 Korea Institute of Science and Technology (KIST), Seoul 02792, Republic of Korea; ‡ Center for Theoretical Physics of Complex Systems, 34998 Institute for Basic Science (IBS), Daejeon 34126, Republic of Korea; § Advanced Analysis and Data Center, Korea Institute of Science and Technology, Seoul 02792, South Korea; ∥ KU-KIST Graduate School of Converging Science and Technology, Korea University, Seoul 02841, Republic of Korea; ⊥ Department of Optical Engineering, 34975 Kumoh National Institute of Technology, Gumi, Gyoungbuk 39235, Republic of Korea; # Quantum Technology Institute, 65408 Korea Research Institute of Standards and Science, Daejeon 34113, Republic of Korea; ∇ Department of Physics, 34995 Pohang University of Science and Technology (POSTECH), Pohang 37673, Republic of Korea; ○ Chemical and Biological Integrative Research Center, Korea Institute of Science and Technology, Seoul 02792, Republic of Korea; □ Division of Bio-Medical Science & Technology, University of Science and Technology (UST), Seoul 01811, Republic of Korea; ■ Department of Physics, Pukyong National University, Busan 48513, Republic of Korea; ▲ Division of Quantum Information, KIST School, Korea University of Science and Technology, Seoul 02792, Republic of Korea

**Keywords:** Diamond, Nanoelectromechanical Systems, Mass Spectrometry, Bacteriophage T4, Multiplexing4

## Abstract

The significance of mass spectrometry lies in its unparalleled ability to accurately identify and quantify molecules in complex samples, providing invaluable insights into molecular structures and interactions. Here, we leverage diamond nanostructures as highly sensitive mass sensors by utilizing a self-excitation mechanism under an electron beam in a conventional scanning electron microscope (SEM). The diamond molecular balance (DMB) exhibits a practical mass resolution of 4.07 MDa, based on its notable mechanical quality factor and frequency stability, along with a broad dynamic range from MDa to TDa. This positions the DMB at the forefront of nanoelectromechanical system (NEMS)-based mass spectrometry operating at room temperature. Notably, the DMB demonstrated its ability to measure the mass of a single bacteriophage T4 by precisely locating the analyte on the device. These findings demonstrate the capability and potential of the DMB as a revolutionary tool for mass spectrometry at room temperature.

## Graphical abstract

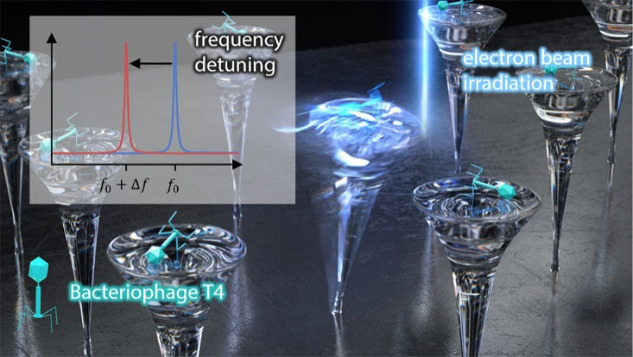

Systematic analysis of molecules in a tissue or cell continues to be pursued with the development of mass spectrometry at the heart of proteomics. The task of identifying and probing diverse molecules is quite challenging because of the inherent complexity and structural diversity of molecular components. Conventional mass spectrometry (MS) can identify species of molecular analytes in the range of 1 to 100 MDa by using soft ionization, electromagnetic fields to manipulate ions, and ensemble averaging of mass-to-charge ratios.^1,2^ In recent years, the limitations of conventional mass spectrometry in terms of mass resolution and dynamic range have prompted the exploration of alternative approaches for mass analysis.

The unprecedented potential of a nanoelectromechanical system (NEMS) for mass sensing has been explored by many research groups.^3–15^ These nanomechanical resonators enable the measurement of individual particle masses by detecting the inertial mass of target analytes regardless of their charge state (ionized or neutral). Initial studies focused on measuring gold nanoparticles and IgM antibody complexes,^3^ followed by investigations involving tantalum nanoclusters,^4,5^ bacteriophage T5,^6^ and the dry mass of bacteria.^7,8^ More recently, NEMS-based mass sensing has been successfully applied to the detection of SARS-CoV-2.^9^ Remarkably, one such system has achieved mass resolution down to the yoctogram scale (a few number of atoms)^10^ while another has demonstrated a broader detection range, extending to gigadalton (GDa) levels, compared to conventional MS.^16^ This extensive dynamic range makes NEMS particularly attractive for mass spectrometry applications, including the analysis of high-mass analytes such as viruses, diverse disease biomarkers, and synthetic nanoparticles for nanomedicine.^6,9^ Despite these advantages, several challenges hinder the widespread adsorption of nanomechanical mass spectrometry. Key limitations include resonance variations due to the mass adsorption location, the mass loading efficiency, the need for cryogenic conditions, and the complexity of both the device and its signal processing. Moreover, the feasibility of analyzing real biological samples remains a critical concern. Ongoing research continues to address these challenges, aiming to enhance the reliability and practicality of NEMS-based MS.^17–20^

Two characteristics of NEMS devices, namely, their minuscule mass and electromechanical degree of freedom, play critical roles in typical NEMS applications. These ingredients are apparent strengths in mass sensing tasks, where the resonant frequency variation of NEMS reflects the mass of adsorbed analytes. A NEMS-based spectrometer can be engineered for specific target mass ranges by adjusting its dimensions, as the frequency-to-mass relationship scales with the resonator’s characteristic dimension, *d* ^–4^. This can be further enhanced by a proper choice of materials.

Diamond is the hardest material on earth and is famous for its durability. With recent improvements in fabrication technology,^21^ its limitations of poor deformability and relatively high brittleness can be lifted at the nanoscale.^22,23^ Furthermore, its outstanding mechanical property, possibly resulting in a high quality factor, is found to be insensitive to environmental temperature,^24^ although its maximum achievable elastic strain and strength are determined primarily by surface conditions of the given structure.^25^ These remarkable characteristics of diamond and fabrication technologies at the nanoscale are about to open a new avenue of diamond NEMS.

In this work, as a proof-of-concept toward room temperature mass spectrometry at the molecular level, we demonstrate the use of a diamond nanostructure as a highly sensitive mass sensor, which we refer to as the diamond molecular balance (DMB). We investigated its structural properties and operating mechanism by conducting experiments in a transmission electron microscope (TEM, Tecnai F20 G2, FEI) and scanning electron microscope (SEM, Quanta 3D FEG, and G4 HX, FEI) with computational support. Calibrated analytes across a wide mass range, from attograms to picograms, were evaluated. The mass sensitivity, along with a wide dynamic range, is attributed to its mechanical quality factor of 236,000, which is relatively high for nanomechanical devices operating at room temperature. The frequency stability of the DMB was assessed to determine its mass resolution resulting from the device alone. Finally, in a seminal validation of its capability, the DMB weighs a single bacteriophage T4 at room temperature by precisely locating the analyte on top of the DMB. The high device integration density and multiplexed measurement capability of the DMB will facilitate a large cross-section of the capture area and high-throughput analysis, significantly enhancing its applicability in fields such as virology and molecular biology.

## Diamond Nanoscale Structure and Self-Oscillation

The DMB was fabricated by applying diamond fabrication techniques (see Supporting Information 1: Fabrication of the Diamond Molecular Balance (DMB) for details).^20^ We used a chemical vapor deposition (CVD) diamond substrate (2.0 × 2.0 × 0.5 mm^3^) of single crystal electronic grade with a ppb level of impurity (element six, Electronic-Grade Single-Crystal Diamonds, ELSC20). A layer of a silicon nitride film was deposited and patterned by using electron beam lithography and a reactive ion etching process. By applying patterned silicon nitride disks as an etch mask, the diamond substrate was then selectively etched. A typical structure for a DMB has dimensions of 600 nm in diameter at the top plateau and 1.5 μm in height. The diameter at the lower bottom of the structure varies within the range of 20 to 60 nm. The crystallinity of the structure after fabrication processes was confirmed by using TEM (see Figure 1c and Supporting Information 2: Transmission electron microscopy (TEM) sample preparationfor details).

**Figure 1:**
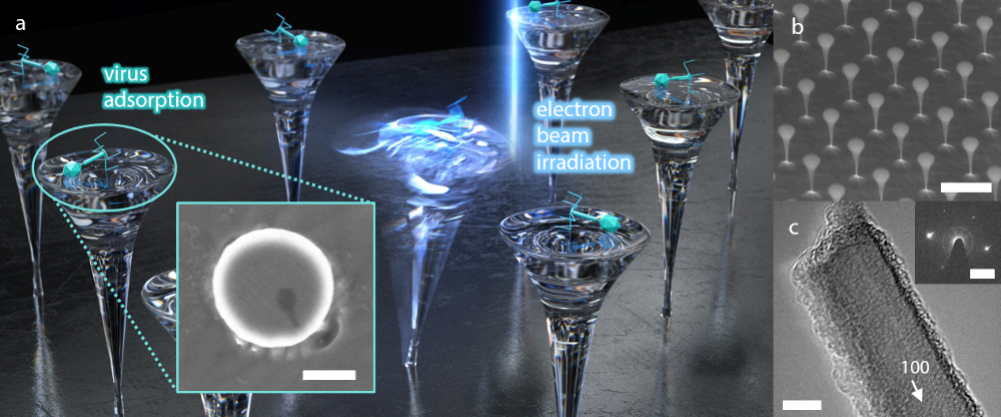
Diamond molecular balances (DMBs). (a) Illustration of DMBs with an incident electron beam. The resonant frequency of the DMB is detuned by the mass of the adsorbed analytes. (inset) A scanning electron micrograph of a bacteriophage T4 virion on top of a DMB (scale bar 300 nm). (b) A SEM image of DMBs (scale bar 2 μm). (c) A TEM image of a DMB (scale bar 5 nm), and (inset) its selected area electron diffraction pattern. The crystallinity of the nanostructure remains well preserved after fabrication processes (scale bar 4 nm^–1^). The white arrow indicates the (100) face of the diamond crystal structure.

The essence of the mass detection mechanism in our DMB lies in the self-oscillation induced by a primary electron beam.^11,26^ Operating in spot mode, a focused electron beam can be accurately positioned in a scanning electron microscope. As primary electrons interact with the diamond nanostructure, secondary electron emission induces positive charging. These localized charges prompt electrostatic interactions with the environment, resulting in nanostructure bending. Under the given charge relaxation conditions, the diamond nanostructure undergoes self-oscillation through cyclic charging and discharging processes.

With its self-oscillation of the DMB, the amplitude of the secondary electron current is periodically modulated as seen in Figure 2a (see Supporting Information 3: Measurement setup for details). The secondary electron signal inherently contains information on the mechanical oscillation at nanoscale as the primary electron beam scans over the DMB during its oscillation. We note the outstanding quality factor (∼236,000) of this system in the frequency domain at room temperature (Figure 2b). This agrees with the observation that the mechanical quality factor in diamond-based nanostructures is relatively insensitive to environmental temperature.^24^ The frequency stability δ*f/f*_0_, based on the Allan deviation as shown in Figure 2c, is assessed to be 3 × 10^–6^, corresponding to the mass resolution of 0.36 MDa at room temperature (see Supporting Information 4: Allan deviation analysis from the time dependent response of the DMB for details).

**Figure 2:**
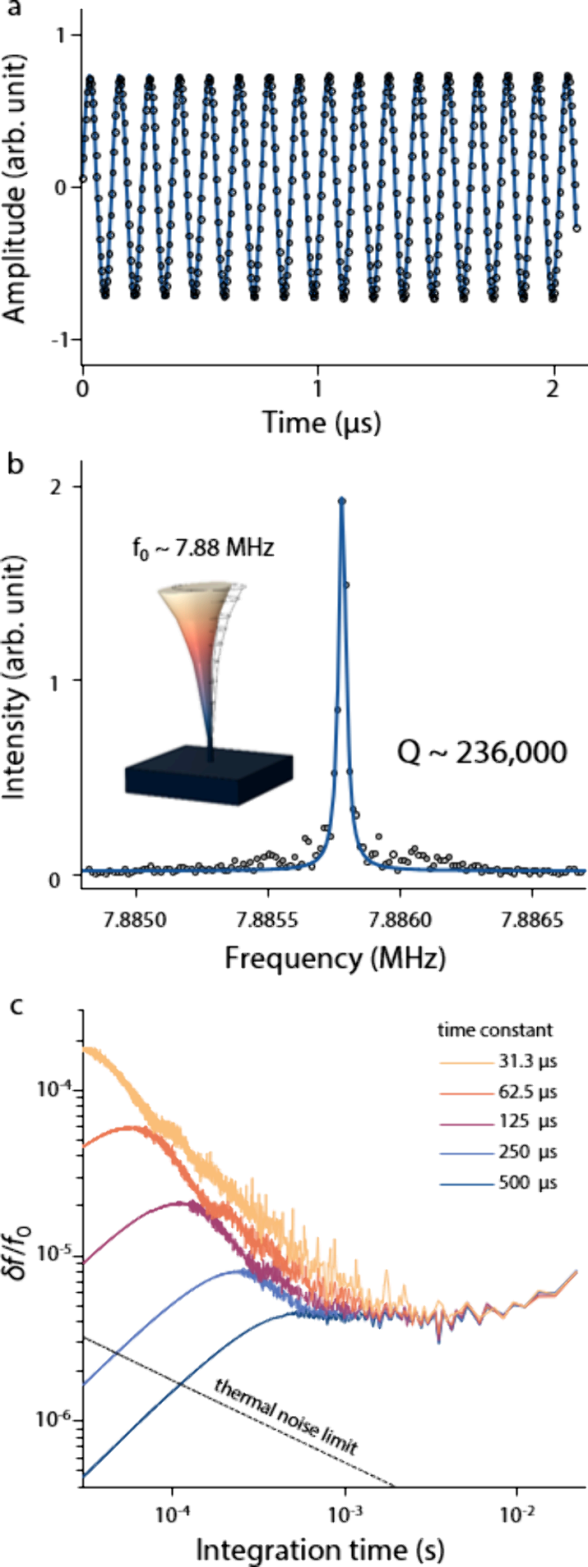
Self-oscillation of DMB and fast Fourier transform analysis. (a) Time-dependent secondary electron intensity resulting from the self-oscillation of the DMB. Secondary electron intensity is periodically modulated by the mechanical motion of the self-oscillating DMB, and the time-dependent intensity signal is well traced by a sinusoidal function. (b) Fast Fourier transform (FFT) result. From the Lorentzian curve fitting, a quality factor of 236,000 was extracted. (inset) Finite element simulation of a resonant mechanical mode of the DMB. The obtained resonance frequency closely matches the finite element calculation result of 7.88 MHz. (c) Frequency stability of the DMB under different time constant conditions. The dashed line indicates the thermal noise limit.

## Ultrawide Mass Detection Range

To explore the mass detection range of DMB, we loaded calibrated mass targets by depositing carbon and platinum composites in different volumes on top of the DMB (Supporting Information 6: Preparation and mass evaluation of calibrated mass analytes for details). The deposited weight was carefully calculated considering the obtained volumes, ratios of constituent elements, and their density information. The obtained frequency detunings are summarized according to loaded weights in Figure 3a. Surprisingly, our DMB demonstrated quantitative mass detection in the range from 1.5 ag to 3.3 pg (0.9 MDa to 2.0 TDa). To date, it measures the widest range (6 orders of magnitude) of masses among the implemented methods for mass detection. The heaviest analyte was nearly 25 times heavier than the effective mass of DMB itself. This wide mass detection range spans from the megadalton (MDa) to the teradalton (TDa) scale. It covers the mass range of various biological objects, including pathogens such as the hepatitis B virus and , which cause transmissible infections (Figure 3a). We note that the spring constant of the DMB stays almost unchanged with the standard deviation of 0.3% over the course of 50 successive mass loading events (Supporting Figure 7).

**Figure 3:**
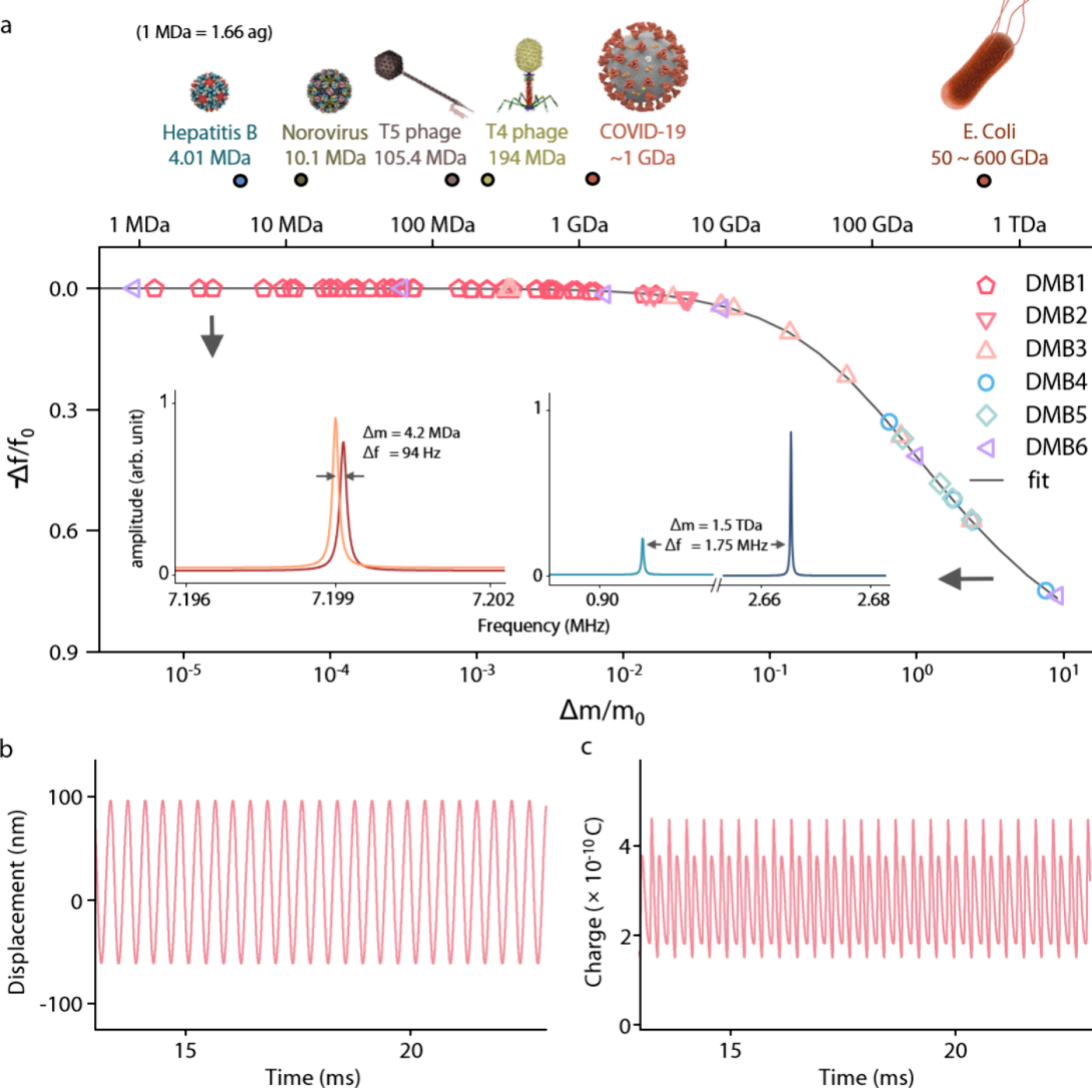
Mass detection range of DMB and a theoretical modeling. (a) (upper) Molecular weights of various viruses and . (bottom) Normalized frequency detunings as a function of normalized weights. The measured frequency detunings from six different DMBs with calibrated mass loading in the range of MDa to TDa (pentagon, inverted triangle, triangle, circle, and diamond; for the leftward triangle see Supporting Figure 4) were traced by numerically obtained results based on the theoretical model (solid line, see Supporting Information 5: Electromechanical modeling of the DMB for details). (inset) Frequency shifted responses with the mass loadings of 4.2 MDa and 1.5 TDa. (b) Time-dependent displacement of DMB. (c) Time-dependent charge state of DMB.

The electromechanical behavior of the DMB can be modeled by a driven-damped harmonic oscillator coupled to charge dynamics induced by an incident electron beam^26^ (see Supporting Information 5: Electromechanical modeling of the DMB for details). The obtained frequency response to mass loading closely matches the numerically obtained results based on the proposed theoretical model. These calculations utilized experimentally obtained parameter values, including charge relaxation time (Supporting Figure 8), resonance frequency, and the quality factor of the DMBs. From the theoretical model, validated by the experimental results, the effective mass of our system as well as frequency detunings at different mass loadings were extracted. We further investigated the time-dependent displacement and charge state of the DMB using parameters obtained from experimental results, as depicted in Figure 3b,c (see Supporting Information 7: Piezoelectrically driven oscillation of the DMB for details). The mechanical motion of the DMB manifests as a limit cycle in phase space (Supporting Figure 11), exhibiting behavior which closely resembles that of a harmonic oscillator.

## Mass Measurement on a Single Bacteriophage T4

As a notable example of the mass measurement of biological analytes at room temperature, we conducted measurements on a single bacteriophage T4. Bacteriophage T4 ( virus T4) is a relatively large, double-stranded DNA virus that infects bacteria. It has a distinctive head-and-tail structure, and a mature phage typically has dimensions of 90 nm in width and 200 nm in length with a weight of approximately 194 MDa (0.322 fg). Metallic layers of Ti and Au, each with a thickness of 5 nm, were deposited on the DMBs to enhance the contrast between the analytes under the electron beam. We applied a calculated volume concentration of the bacteriophage T4 solution to facilitate the adsorption of analytes on the surface of the DMB (see Supporting Information 9: Preparation of bacteriophage T4 in solution for details). The adsorbed bacteriophage T4 virions were neatly positioned on the DMB, clearly distinguishable from the top plateau surface. Almost no residual substances, including salt and buffer materials, were observed in the surface elemental analysis, suggesting effective rinsing during sample preparation (Supporting Figure 14). During the adsorption process, the structure of the analyte remained intact as evidenced by the TEM image, revealing the detailed body structure of bacteriophage T4 including its tail fibers (Figure 4d). It was demonstrated that DMB can accurately measure the inertial mass of bacteriophage T4 virions (Figure 4a). A distinct resonance peak shift, corresponding to a mass of 184 MDa, was observed in the frequency spectrum for a single bacteriophage T4 (Figure 4a).

**Figure 4:**
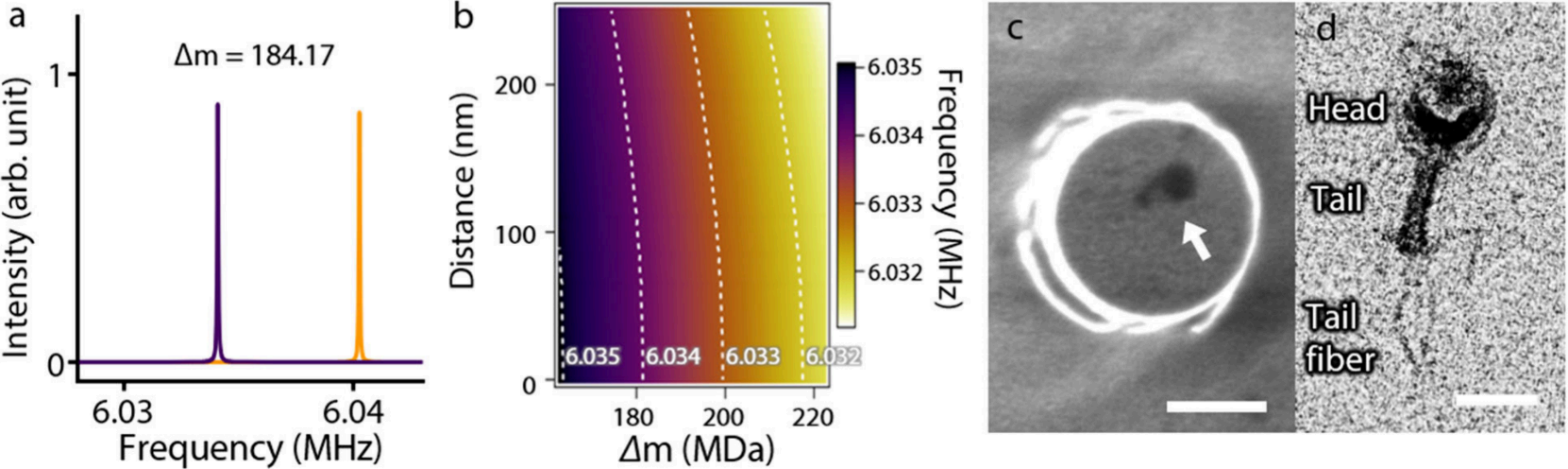
Mass measurement on a single bacteriophage T4. (a) Detuned frequency response of a DMB before (yellow) and after (purple) loading a single bacteriophage T4. (b) Corrections on frequency detunings as a function of varying positions along the moving direction, the distance from the axis perpendicular to the moving direction, and masses of analytes on the plateau of the DMB. The dashed lines on the plot represent equal values of correction on the frequency detuning. (c) Scanning electron micrograph image (top view) of a single bacteriophage T4 on top of the DMB (scale bar 300 nm). (d) Transmission electron micrograph image of a bacteriophage T4, revealing detailed body structure including tail fibers (scale bar 100 nm).

To address the position dependence of the frequency response, we calculated corrections for frequency detunings as a function of varying positions along the moving direction, the distance (*d*) from the axis perpendicular to the moving direction, and masses of analytes on the plateau of the DMB (see Figure 4b and Supporting Information 10: Frequency compensation based on the analyte’s position on the top plateau of the DMB for details). The analyte position on the DMB is corrected using the relation

1 $$\Delta m_{eff}\left(d\right)=\Delta m_{p}f\left(d\right)$$

where Δ*m*_eff_(*d*) is the effective mass of the analyte measured by the DMB, Δ*m*_p_ is the actual mass of the analyte, and *f*(*d*) is a correction factor that accounts for its position on the DMB (see Supporting Information 10: Frequency compensation based on the analyte’s position on the top plateau of the DMB for details).

A mass resolution of 4.07 MDa was achieved at room temperature with adsorption–desorption noise and the positional information limited by the spatial resolution (approximately 10 nm) of the scanning electron microscope. A 10 nm SEM resolution results in mass errors of 0.015 MDa at the center and 0.55 MDa at the edge (250 nm) for a 200 MDa analyte as shown in Supporting Figures 15 and 20 (see Supporting Information 11: Mass resolution of the measurement with the DMB for details).

As seen in the SEM image, the analyte exhibits a finite contact area rather than a point contact. Additionally, the stiffness of the analyte results in a downward shift and broadening of the resonance peak.^6,27,28^ However, finite element simulations have shown that the impact of these factors is negligible compared to other determinants of mass resolution (see Supporting Information 11: Mass resolution of the measurement with the DMB for details). This could be attributed to the minimal displacement induced by the DMB. The mass resolution is primarily degraded by the adsorption–desorption noise.^29,30^ This issue can be mitigated by adopting an in situ analyte loading system^31,32^ that maintains an optimal vacuum level, thereby minimizing adsorption–desorption noise. The calculated mass from frequency detuning shows a slight discrepancy from typical estimates. The analyte is most likely to be an immature bacteriophage within the sample batch.^33^ The discrepancy observed in our measurements may reflect the loss of Hoc/Soc decorations (<14 MDa)^34^ or partially filled capsids (3–10 MDa)^35–37^ reported in prior structural studies. Measurements from other DMBs yielded slightly varying mass values, all near the typical mass of 194 MDa (Supporting Figure 16). There have been instances where measurement was taken with two or more bacteriophage T4 virions simultaneously on a single DMB, resulting in mass values that are multiples of the typical mass.

## Discussion

As demonstrated, DMB effectively addresses the key limitations of NEMS-based mass spectrometry. The outstanding mechanical quality factor (∼236,000) of the DMB enhances frequency stability and minimizes signal-to-noise degradation, achieving sub-MDa mass resolution. With its well-defined inverted cone-shaped nanostructure, the DMB ensures that the target analyte adheres exclusively to the top surface, preventing adsorption on the sidewalls and thereby minimizing resonance degradation. Additionally, the SEM-based measurement technique enables the correction of resonance frequency shifts through precise analyte positioning. The use of a diamond nanostructure array enhances analyte capture efficiency by providing a high-density surface for adsorption, improving detection sensitivity and throughput (see Supporting Information 13: Cross section of the DMB array for details).

Beyond resolving these fundamental limitations, DMB introduces significant advancements that further expand its capabilities. While conventional NEMS-based mass spectrometry devices typically require low-temperature environments to achieve high mechanical quality factors, the DMB operates at room temperature, eliminating the need for a cryogenic apparatus. This not only streamlines experimental setups but also makes the DMB particularly advantageous for studying molecular interactions under pathological conditions. Its remarkable mass detection range—from MDa to TDa—represents a significant advancement in next-generation mass spectrometry, addressing critical gaps in existing weighing technologies. In addition, its ability to accurately measure individual biological specimens underscores its potential as a powerful tool for molecular-level investigations (Table 1).

**Table 1:** Comparison of NEMS-MS Platforms in Terms of Biological Applicability and Target Mass Range

Targets	Demonstrated Mass Range	Temperature	Reference
T4 bacteriophage	0.9 MDa to 2.0 TDa	Room temperature	This work
BSA	10 kDa to hundreds of MDa	∼40 K	^38^
IgG, IgM, BSA	1 kDa to 1 MDa	Room temperature	^13^,^14^,^39^
*E. coli*	6–200 GDa	Room temperature	^28^
T5 bacteriophage	25–106 MDa	Room temperature	^6^
IgM	0.5–15 MDa	70, 140 K	^3^
Tantalum cluster	70 kDa to 4.5 MDa	77 K	^4^,^5^
	0.7–763 fg (0.42–460 GDa)	Room temperature	^7^,^8^
SARS-COV-2	3.3–500 MDa	Room temperature	^9^
Few atom	1.7 yg	4.3 K	^10^

The DMB’s high device integration density, achieved through a top-down fabrication approach, enhances its scalability without the need for Supporting circuitry. The implementation of multiplexed measurements on integrated DMB arrays, combined with precise electron beam positioning, opens new possibilities for high-throughput analysis (Figure 5). The combination of multiplexed measurement equipped with an in situ analyte loading technique, such as a micro injection system,^31,32^ presents a potential avenue for advanced mass spectrometry with an ultrawide mass detection range at room temperature. By overcoming the fundamental limitations of NEMS-based mass spectrometry and integrating its own groundbreaking technological advancements, the DMB serves as a benchmark for molecular-scale mass spectrometry, unlocking new possibilities in virology, molecular biology, and nanomedicine.

**Figure 5:**
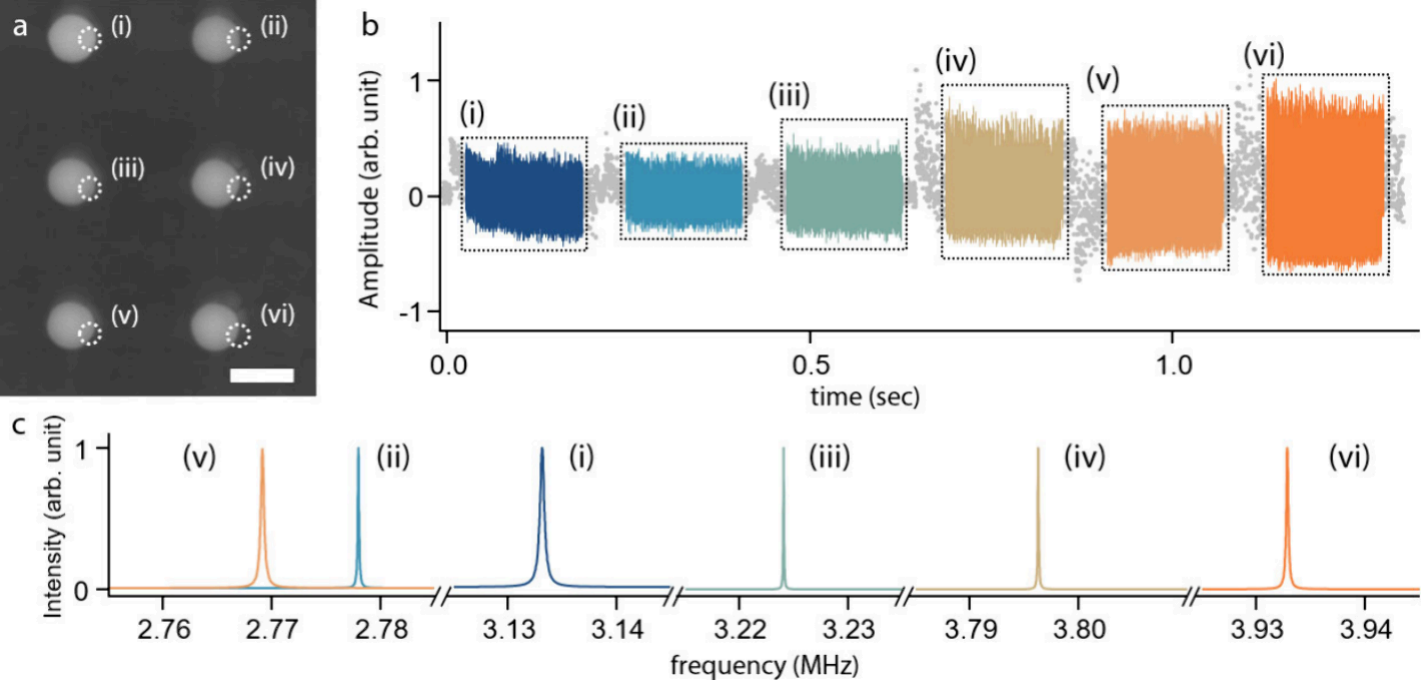
Time-division multiplexed measurement on an array of DMBs. (a) Scanning electron micrograph image of the DMBs (scale bar 1 μm). Locations where the electron beams were directed are indicated by dotted circles. (b) Time-dependent response of DMBs. Portions of the real-time data, sequentially obtained from different DMBs (i–vi), are used for FFT analysis. (c) FFT results obtained from the DMBs (i–vi).

## Supplementary Material



## Data Availability

All data needed to evaluate the conclusions in the paper are present in the paper or the Supporting Information.

## Notes

Additional text and figures as described in the text (PDF)
