# Supplementary material for: Diamond Molecular Balance: Ultra-Wide Range Nanomechanical Mass Spectrometry from MDa to TDa
Source: Nano Lett. 2025 Jun 19;25(26):10497–503. doi: 10.1021/acs.nanolett.5c02032 (PMC12232386; doi:10.1021/acs.nanolett.5c02032)
Supplement: Supplementary file 1 [file nl5c02032_si_001.pdf]

Diamond Molecular Balance: Ultra-Wide Range Nanomechanical Mass Spectrometry from MDa to TDa

Donggeun Lee[1,+], Seung-Woo Jeon[1,+], Chang-Hwan Yi[2,+], Yanghee Kim[3], Yeeun Choi[1,4], Sang-Hun Lee[5], Jinwoong Cha[6], Seung-Bo Shim[6], Junho Suh[7], Il-Young Kim[1], Dongyeon Daniel Kang[1], Hojoong Jung[1], Cherlhyun Jeong[8,9], Jae-pyoung Ahn[3], Hee Chul Park[10,], Sang-Wook Han[1,4,11,*], Chulki Kim[1,*]*

[1]Center for Quantum Technology, Korea Institute of Science and Technology (KIST), Seoul 02792, Republic of Korea

[2]Center for Theoretical Physics of Complex Systems, Institute for Basic Science (IBS), Daejeon 34126, Republic of Korea

[3]Advanced Analysis and Data Center, Korea Institute of Science and Technology, Seoul 02792, South Korea

[4]KU-KIST Graduate School of Converging Science and Technology, Korea University, Seoul 02841, Republic of Korea

[5]Department of Optical Engineering, Kumoh National Institute of Technology, Gumi, Gyoungbuk 39235, Republic of Korea

[6]Quantum Technology Institute, Korea Research Institute of Standards and Science, Daejeon 34113, Republic of Korea

[7]Department of Physics, Pohang University of Science and Technology (POSTECH), Pohang 37673, Republic of Korea

[8]Chemical and Biological Integrative Research Center, Korea Institute of Science and Technology, Seoul 02792, Republic of Korea

[9]Division of Bio-Medical Science & Technology, University of Science and Technology (UST), Seoul, Republic of Korea

[10]Department of Physics, Pukyong National University, Busan 48513, Republic of Korea.

[11]Division of Quantum Information, KIST School, Korea University of Science and Technology, Seoul 02792, Republic of Korea

[+]These authors contributed equally: Donggeun Lee, Seung-Woo Jeon, Chang-Hwan Yi

[*]These authors jointly supervised this work: Chulki Kim, Sang-Wook Han, Hee Chul Park.

[*]e-mail address:chulki.kim@kist.re.kr, swhan@kist.re.kr, hcpark@pknu.ac.kr

KEYWORDS: Diamond, Nano-Electro-Mechanical Systems, Mass Spectrometry, Bacteriophage T4, Multiplexing4

Supporting Information 1: Fabrication of the Diamond Molecular Balance (DMB)

To fabricate the diamond inverted nanocone structure, a top-down diamond fabrication process was employed. We purchased a chemical vapor deposition (CVD) diamond substrate (2.0 x 2.0 x 0.5 mm^3) in single crystal electronic grade with ppb level of impurity (Element six, Electronic-Grade Single-Crystal Diamonds, ELSC20). Initially, a 300 nm thick silicon nitride layer was deposited on the diamond substrate using plasma-enhanced chemical vapor deposition (PECVD). Subsequently, electron beam lithography was used to pattern 600 nm in diameter dots on this layer. Following this, a reactive ion etching (RIE) process was utilized to etch the silicon nitride, and the remaining resist was removed through a piranha cleaning process (H_2SO_4: H_2O_2 = 3:1) at 80°C. Using the silicon nitride layer as a hard mask, O_2 inductively coupled plasma (ICP) RIE was employed to vertically etch the diamond to a depth of approximately 300 nm. Using an angled plasma etching process with a designed Faraday cage, as shown in Supporting Fig. 1, an inverted nanocone structure was realized with an upper diameter of approximately 600 nm, a lower neck diameter ranging from 20 to 60 nm, and a height of 1.5 µm. The remaining silicon nitride was subsequently removed using an HF solution.

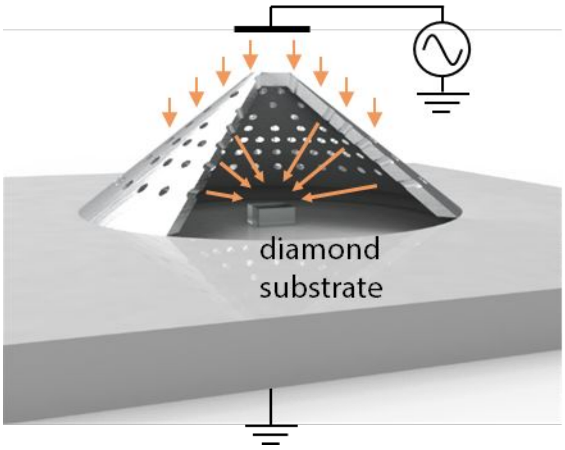

Supporting Figure 1. For the angled oxygen plasma etching process, we used a designed Faraday cage made of stainless steel with a conical shape and perforations. The oxygen plasma is generated on the cage because it is electrically grounded.

Supporting Information 2: Transmission electron microscopy (TEM) sample preparation

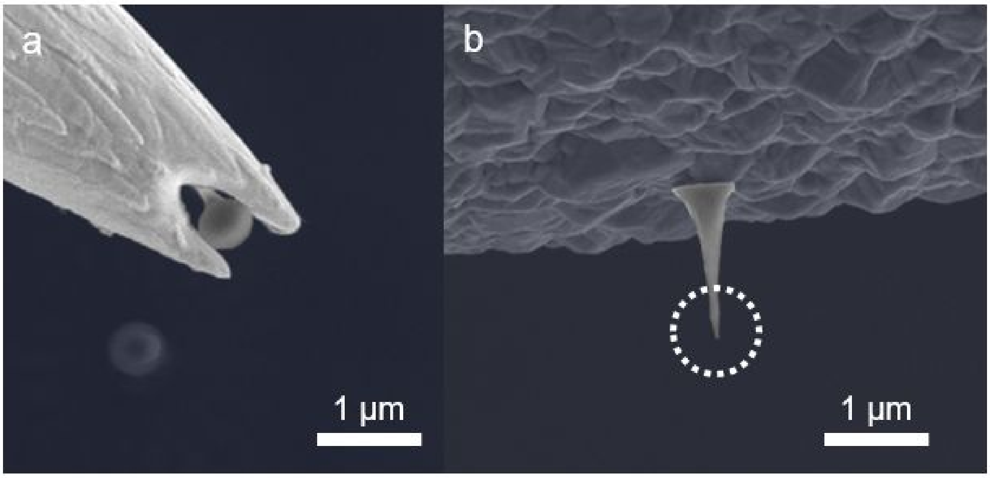

Supporting Figure 2. TEM images of the physically fractured DMB. A fork-shaped tungsten manipulator was fabricated using a focused ion beam (FIB) and used it to fracture the DMB from the substrate. The detached part of the DMB was successfully transferred onto a copper grid using the manipulator, facilitating TEM analysis.

Using a focused ion beam (FIB), tungsten tips can be shaped into desired configurations. By employing FIB milling, a tungsten tip can be precisely sculpted into a fork structure by utilizing a manipulator (MM3E, Kleindiek) installed within the chamber. Leveraging this capability, we physically fractured diamond nanocone structures and affixed them onto a copper grid to further investigate their material properties. Subsequently, using Transmission electron microscopy (TEM, Tecnai, FEI), we examined the crystallinity of the fractured portion, the thinnest section in the DMB.

Supporting Information 3: Measurement set-up

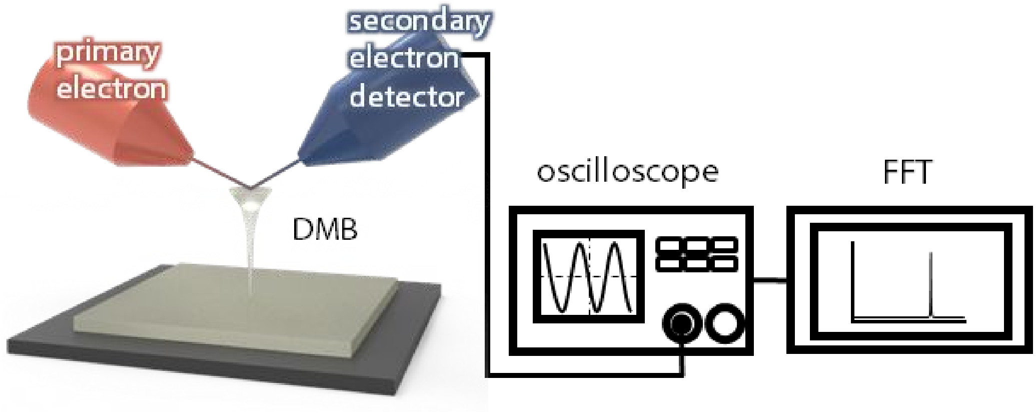

Supporting Figure 3. Illustration of measurement set-up. By irradiating primary electrons in spot mode, the spot area exposed to the electron beam becomes positively charged. These localized charges prompt electrostatic interactions with the environment, resulting in nanostructure bending. Under given charge relaxation conditions, the diamond nanostructure undergoes self-oscillation through cyclic charging and discharging processes. With its self-oscillation of the DMB, the amplitude of the secondary electron current is periodically modulated. The secondary electron signals are monitored and recorded in an oscilloscope. The frequency responses of the DMB are collected after performing a Fast Fourier Transform (FFT) on this data.

Supporting Information 4: Allan deviation analysis from the time dependent response of the DMB

We analyze the frequency stability of our DMB system by examining the Allan-deviation (AD). Our approach utilizes the experimentally obtained time-dependent signal from the secondary electron detector, represented as the real-valued signal $s(t_n)$, where t_n denotes the discrete time sequence of measurements with the time interval $t_i - t_{i-1} = \Delta t = 4$ ns.

1. **Analytic signal from the real-valued time-dependent amplitude signal**

From the real-valued signal $s(t)$, we will deduce the instantaneous frequency for analysis by constructing the analytic signal[1]

$$z(t) = s(t) + i\mathrm{HT}[s(t)]\ , \qquad (1)$$

where $i = \sqrt{-1}$, and $\mathrm{HT}[s(t)]$ represents the Hilbert transform that is the time-domain convolution of $s(t)$ with the function $w(t) = \frac{1}{\pi t}$, namely $\mathrm{HT}[s(t)] = \mathrm{p.v.}\int_{-\infty}^{\infty} \frac{s(\tau)d\tau}{\pi(t-\tau)}$. The Fourier transform $\mathcal{F}[\,\cdot\,]$ of $w(t)$ from the time domain (t) to the frequency domain (f) is given as

$$\mathcal{F}[w(t)] = -i\,\mathrm{sgn}(f) = \begin{cases} -i = e^{-\frac{\pi i}{2}}, & if\ \ f > 0 \\ 0, & if\ \ f = 0 \\ i = e^{\frac{\pi i}{2}}, & if\ f < 0 \end{cases}. \qquad (2)$$

This implies a $\pm\frac{\pi}{2}$ phase shift in the f-domain such that

$$\mathcal{F}\{\mathrm{HT}[s(t)]\} = -i\,\mathrm{sgn}(f)\cdot\mathcal{F}\{s(t)\} \qquad (3)$$

$$\Rightarrow i\mathrm{HT}[s(t)] = \mathcal{F}^{-1}[\mathrm{sgn}(f)\cdot\mathcal{F}\{s(t)\}] \qquad (4)$$

$$\Rightarrow \mathcal{F}[z(t)] = \overbrace{\mathcal{F}[s(t)]}^{even} + \overbrace{\mathrm{sgn}(f)\cdot\mathcal{F}[s(t)]}^{odd} \qquad (5)$$

Accordingly, the Fourier transform of the analytic signal contains only the positive frequency components:

$$Z(f) = \begin{cases} 2S(f), & if\ f > 0 \\ S(f), & if\ f = 0, \\ 0, & if\ f < 0 \end{cases} \qquad (6)$$

where $Z(f) = \mathcal{F}[z(t)]$ and $S(f) = \mathcal{F}[s(t)]$.

2. **Instantaneous phase and frequency from the analytic signal**

After constructing the analytic signal, we can extract the instantaneous phase $\phi(t)$ as well as the instantaneous frequency $f(t)$ straight forwardly by obtaining the phase of the complex-valued analytic signal $z(t) = |z(t)|e^{i\phi(t)}$

$$\phi(t) = -i\ln\left(\frac{z(t)}{|z(t)|}\right) \qquad (7)$$

and its time derivative

$$f(t) = \frac{d\phi(t)}{dt}. \qquad (8)$$

3. **Instantaneous phase and frequency with FFT**

Based on the above formal reasoning, we deduce the instantaneous phase and frequency using the Fast-Fourier-Transform (FFT) algorithm. First, we transform the original signal $s(t_n)$ into the frequency domain $S(f_n)$ by applying FFT. We then remove the negative frequency component ($f_n < 0$) and double the positive frequency components [$2S(f_n)$ for $f_n > 0$]. Subsequently, we perform the inverse FFT (IFFT) to construct the analytic signal $z(t_n)$.

The calculation is carried out in a single step by performing FFT in a real-to-complex manner with a multiplying factor of 2, followed by IFFT in a complex-to-complex manner. In practice, we implement this numerical method using the FFTW3 Fortran library[2]. From the constructed analytic signal $z(t_n)$,

we extracted the instantaneous phase $\phi(t_n) = -i\ln(z(t_n)/|z(t_n)|)$ along the discrete time sequence t_n. Then, the discrete-time instantaneous frequency

$$f(t_n) = \frac{d\phi(t_n)}{dt} \approx \frac{\Delta\phi(t_n)}{\Delta t} \qquad (9)$$

is calculated.

After taking the FFT, the frequency sampling bandwidth Δf around the nominal frequency f_0 (main peak frequency) is predetermined before performing the IFFT. Here, we emphasize that our DMB operates based on a self-sustaining oscillation mechanism, where there is no driving external excitation with a specific frequency. In this mechanism, the main peak frequency f_0 of the self-oscillation itself serves as the nominal frequency. We also stress that the frequency window Δf primarily regulates the noise contributions in the computed instantaneous frequency fluctuation. A narrower window implies that fewer noise factors are included in the constructed instantaneous frequency.

4. **Frequency Allan deviation**

We are now prepared to assess frequency stability by computing the Allan deviation (AD). In our approach, we implement the non-overlapped Allan deviation. To begin with, we divide the discrete time series of the instantaneous frequency $f(t_n)$ into N non-overlapping intervals, each with an equal integration time $\tau = M\Delta t$, where M is the number of sampling point of time in each interval. Within each interval k ($1 \leq k \leq N$), we compute the average frequency:

$$\langle f_k(\tau)\rangle = \frac{1}{\tau}\int_{(k-1)\tau}^{k\tau} f(t)dt = \frac{1}{\mathrm{M}\Delta t}\sum_{j=1}^{M} f[(k-1)\tau + j\Delta t]\Delta t. \qquad (10)$$

According to the definition of the fractional frequency Allan deviation,

$$\sigma_A(\tau) = \frac{df}{f_0} = \frac{1}{f_0}\sqrt{\frac{1}{2}\frac{1}{(N-1)}\sum_{k=1}^{N-1}(\langle f_{k+1}\rangle - \langle f_k\rangle)^2} \qquad (11)$$

we obtain the frequency stability range df which is confirmed as ~ 30 Hz in our DMB.

The Allan deviation is a robust measure of frequency stability and effective in identifying noise characteristics in the experimental signal[3]. In our computations, the noise analysis can be carried out by controlling the sampling band with Δf in the IFFT stage.

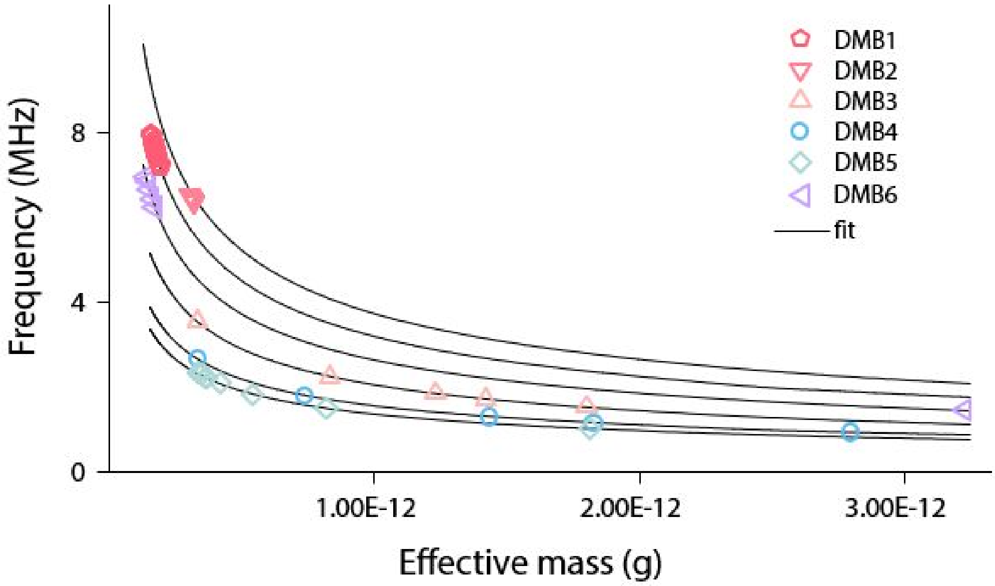

Supporting Figure 4. Resonance frequencies for six DMBs with different effective masses.

Supporting Information 5: Electromechanical modeling of the DMB

1. Theoretical model of the DMB

We analyze the experiment results by simplifying the DMB as an inverted-cone-shaped oscillator in which the cross-sectional area is $A(z)$ and height is l. We aim to understand the self-oscillation of the DMB initiated by an incident electron beam. In such a system with a large aspect ratio of its height and its cross-section area, $\frac{l}{\sqrt{A(z)}} \gg 1$, as those found in typical nanomechanical setups, shear forces can be safely disregarded so that we can apply the Euler-Bernoulli theory with a pure torque to compute the transverse motion of the DMB.

The dynamics of the transverse motion is given by the displacement $u(z, t)$ along the longitudinal axis z and are described by the following equation:

$$\rho A(z)\frac{\partial^2 u}{\partial t^2} + \frac{\partial^2}{\partial z^2}\left[EI(z)\frac{\partial^2 u}{\partial z^2}\right] - p_0\frac{\partial^2 u}{\partial z^2} = 0, \quad (12)$$

where $I(\mathrm{z})$ is the moment of inertia, E is the Young's modulus, ρ is the density, and the impact of axial stress, p_o, from fabrication. The solutions of the spatiotemporal displacement $u(\mathrm{z,t})$ are separable and provide a complete set of the orthonormal basis;

$$u(z,t) = \sum_n \phi_n(z)\chi_n(t), \quad (13)$$

Note that the spatial solution $\phi(z)$ satisfies a single-clamped boundary condition as follows:

$$\phi(0) = \left.\frac{d\phi}{dz}\right|_{z=0} = \left.\frac{d^2\phi}{dz^2}\right|_{z=l} = \left.\frac{d^3\phi}{dz^3}\right|_{z=l} = 0, \quad (14)$$

where the third and fourth conditions come from the requirement for zero transverse force and zero torque at the free end.

Taking all those dynamical factors into account, the governing equation of motion for the DMB is expressed as follows:

$$\rho A(z)\frac{\partial^2 u}{\partial t^2} + \frac{\partial^2}{\partial z^2}\left[EI(z)\frac{\partial^2 u}{\partial z^2}\right] + \Gamma\frac{\partial u}{\partial t} - p_0\frac{\partial^2 u}{\partial z^2} - f_c(u;t) = 0. \quad (15)$$

The cross-sectional area of the DMB is subject to axial forces or loads (tensile or compressive). The constant axial force per unit area, or stress p_o, which modifies the natural frequencies, is generated during the fabrication process

To simplify Eq. (15), we integrate it with respect to z by multiplying the displacement $\phi_m(z)$ with an assumption that the curvature of the DMB is slowly varying. We can further simplify it by defining the effective mass M_m for each resonance mode,

$$M_m = \rho \int_0^l A(z)\phi_m^2 dz, \quad (16)$$

showing the collective effect of the displacement distribution on dynamics. The mode frequency is determined by

$$\omega_{m0}^2 = \frac{E}{M_m}\int_0^l \phi_m(z)(I(z)\phi_m''(z))''dz - \frac{p_0}{M_m}\int_0^l \phi_m(z)\phi_m''(z)dz, \quad (17)$$

where the integration is performed over z, accounting for the curvature of the nanostructure. This approach, which necessitates precise mechanical analysis possibly using COMSOL, simplifies the understanding of the dynamics of the DMB, manifesting the importance of the effective mass in resonance characteristics.

2. Electrical interactions and capacitive forces

Since the DMB is positively charged under the irradiation of the e-beam and electrostatically coupled with an environmental ground plate, the model with a simple capacitor approximation is good enough to describe the effective force acting on our DMB. Here, the derivative of electrostatic energy,

$$U(u;t) = \frac{1}{2}CV^2 = \frac{q(t)^2}{2C(u)}, \quad (18)$$

with respect to the displacement acts as an electrostatic force. The capacitance,

$$C(u) \cong \int_0^l \frac{\varepsilon_o A(z)}{d+u(z)} dz, \quad (19)$$

is defined as a function of displacement.

Note that the integral for the capacitance is computed along the curved path. The electrostatic force can be approximated by the following

$$F_c(q;t) \approx -C_0\left(\int_0^l dz \sum_n (\phi_m \phi_n + \phi_m^{'} \phi_n^{'})\chi_n\right)V^2. \quad (20)$$

3. Numerical calculation of the self-oscillation

Let us consider the first mode to describe the dynamics of the DMB. Eq. (15) transforms to a general driven-damped coupled harmonic oscillator as follows:

$$\ddot{x} + \gamma\dot{x} + \omega_0^2 x = F_c(q;t). \quad (21)$$

In the following, we elaborate on the detailed modeling procedures employed to generate the results presented in the main manuscript. To begin with, we transform the second-order equation in Eq. (21) into a set of coupled first-order dynamical equations, as outlined below:

$$\dot{x} = y \quad (22)$$
$$\dot{y} = -\gamma y - \omega_0^2 x + F_c(q;t)\,. \quad (23)$$

Here, the damping ratio $\gamma = \frac{\omega_0}{Q}$ is fixed with a constant quality factor $Q = 2 \times 10^5$, and the charge q-induced self-oscillating driving force is given as

$$F_c(q;t) = \alpha q(t)^2 \quad (24)$$

for $\alpha = -\frac{1}{M_0}\frac{1}{2C^2}\frac{dC}{dx}$; $C = \frac{C_0}{|x - x_c|}$ and $\frac{dC}{dx} = 3 \times 10^{-2}$. Note that the initial frequency ω_0 and the effective mass M_0 are free parameters provided by the experiment or the first principal numerical simulations.

Next, we model the charge dynamics for the DMB as follows:

$$\dot{q} = -q\left(\frac{|x - x_c|}{RC_0} + \frac{\delta i_b^g}{Q_{max}}\right) + \delta i_b^g\,, \quad (25)$$

where we assume the electron beam current following the Gaussian distribution;

$$i_b^g = i_b e^{-\left(\frac{x - x_c^g}{\sqrt{2}\sigma}\right)^2}, \quad (26)$$

with $\sigma = 1$ with $x_c^g = 3.5$ and $x_c = 0$ denoting the shifted zero-point center, and $i_b = 4 \times 10^{-9}$. The

main charge time factor $\frac{1}{RC}$ is obtained with a constant

$$R \times C = \tau_{RC} = 0.9\ \mu\mathrm{s} \qquad (27)$$

while the remaining terms, δ and Q_{max}, are selected as 9.23×10^{-2} and 1×10^{4}, respectively.

The value of τ_{RC} was obtained experimentally as shown Supporting Fig. 8, while C was calculated using Eq. 20 to be approximately $\sim 5.4 \times 10^{-20}$ [C]. Along with τ_{RC} and C, R was calculated to be $\sim 1.7 \times 10^{13}$ [Ω].

After setting up all the necessary pre-fix parameters, the time propagation of variables $(x,\ \dot{x},\ q,\ \dot{q})$ is performed by implementing the fourth-order Runge-Kutta method. Then, the obtained time-dependent displacement is Fourier transformed to see the peak positions: the mode frequency ω.

The motion of the DMB reaches a limit cycle in the (position, momentum) phase space after sufficient propagation time, as illustrated in Supporting Fig. 10. It turns out that the trajectory of this limit cycle maintains a sinusoidal behavior at the fundamental resonance frequency ω_0, described by $x(t) \approx x_0 + a\ cos(\omega_0 t)$. This implies that while the self-oscillating behavior is robust against perturbations, the frequency shift is sensitive to mass variations. Based on this finding, the relationship between mass variations and the self-oscillating frequency can be effectively approximated by simple harmonic oscillation.

The procedure is carried out repeatedly by adding up an incremental mass sequentially, $M_0 \rightarrow M_0 + \Delta M$, to deduce the incremental mass-dependent frequency shift: $\omega_0 \rightarrow \omega_0 + \Delta\omega$. The system-independent universal behavior of the relation $(\Delta\omega, \Delta M)$ is also confirmed by observing the scaled one as $\left(\frac{\Delta\omega}{\omega_0}, \frac{\Delta M}{M_0}\right)$. Figure 3a in the main manuscript clearly shows the strong agreement between the theoretical model calculations and the experimental results.

Supporting Information 6: Preparation and mass evaluation of calibrated mass analytes

Calibrated mass analytes were prepared by utilizing a dual beam microscope (Scanning Electron Microscope/Focused Ion Beam, FEI Quanta 3D). The calibrated mass analytes were deposited by irradiating the desired area with an electron beam[4]. For larger mass analytes, a Pt-conjugated carbon composite gas was injected while irradiating the area with an electron beam. To quantify the mass of the calibrated mass analytes, we measured the volume of the target using atomic force microscopy (AFM, XE-100, Park Systems) for height measurement and SEM imaging for areal measurement. Additionally, the compositional ratio of the deposited materials was measured using Energy-Dispersive Spectroscopy (EDS). Using this information, we calculated the exact mass of the analytes.

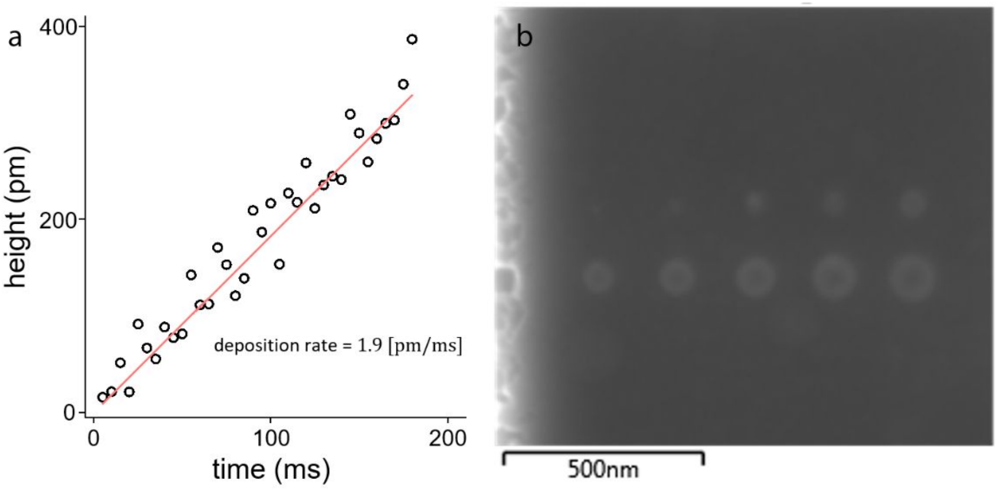

Supporting Figure 5. Preparation and mass evaluation of calibrated mass analytes. a. Height measurement result as a function of deposition time. b. SEM image of deposited carbon islands with varying areas.

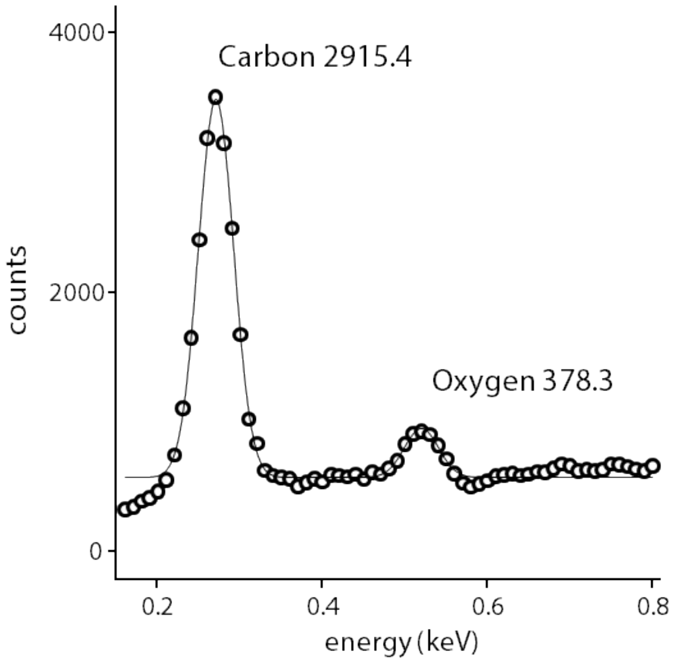

Supporting Figure 6. EDS result of the deposited carbon composites. The ratio between carbon and oxygen was 88.5:11.5.

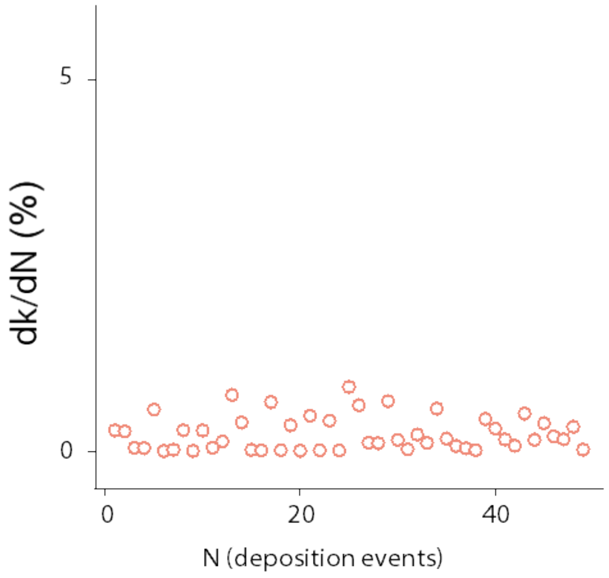

Supporting Figure 7. Spring constant variation with sequential electron beam induced deposition (EBID) events.

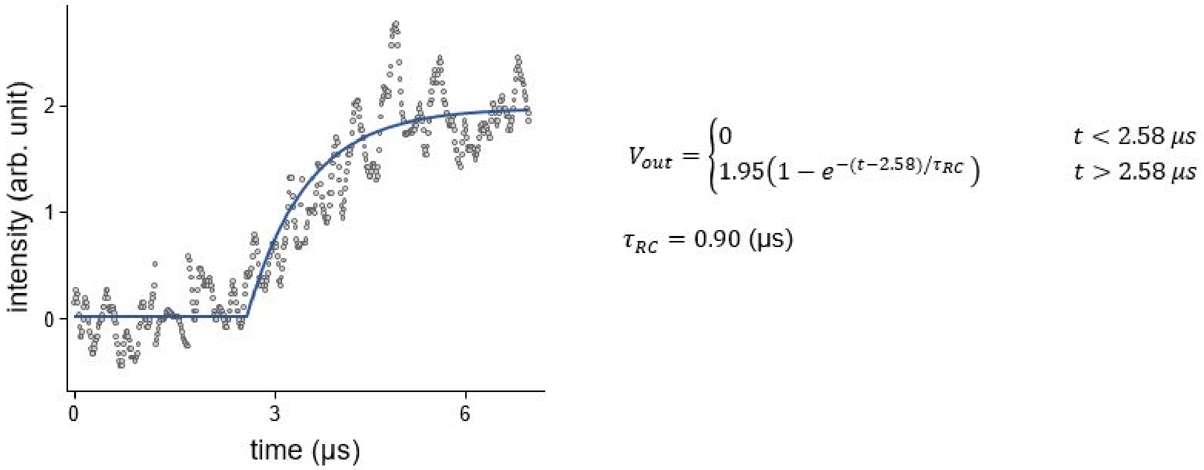

Supporting Figure 8. Transient response to the incident electron beam (circle: data, and line: fitted result). RC time constant of 0.90 μs, associated with charging, can be derived from the transient response.

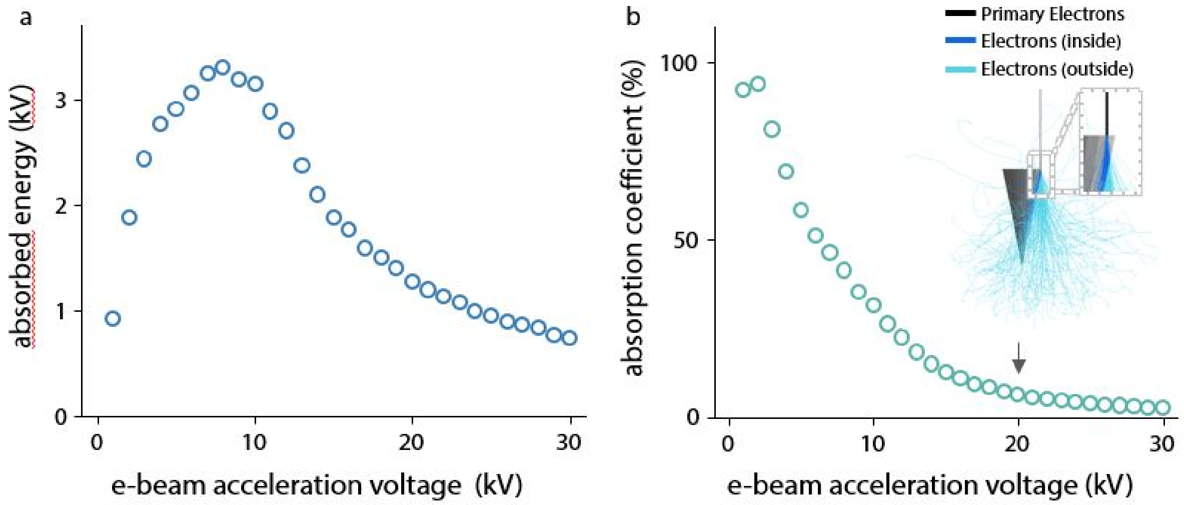

Supporting Figure 9. The Monte Carlo simulation results for absorbed energy and absorption coefficient with incident electrons on a DMB using CASINO v3.3.0.4[5]. **a** Absorbed energy at different acceleration voltages. **b** Absorption coefficient at different acceleration voltages. Inset shows the trajectories of 1000 electrons on a DMB. In the experiment we used the acceleration voltage of 20 kV, which corresponds to an absorption coefficient of 6.8 %.

Supporting Information 7: Piezoelectrically driven oscillation of the DMB

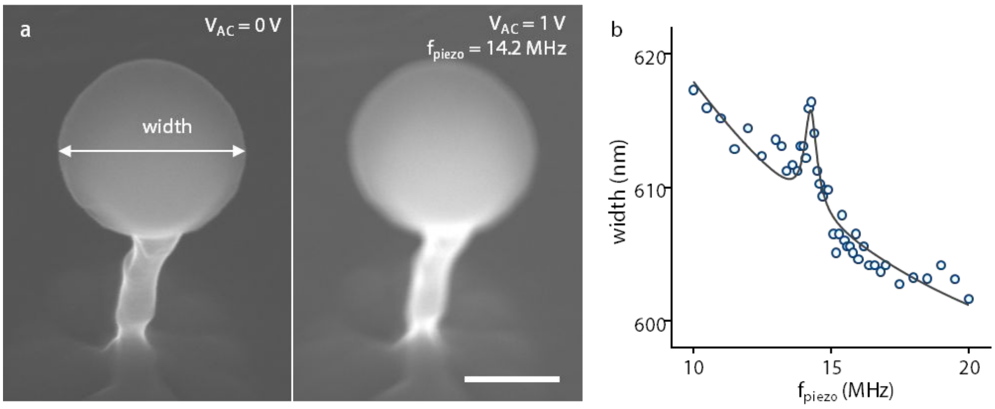

Supporting Figure 10. a Scanning electron micrograph images of the DMB at different amplitudes of the actuation voltages of 0 V (left) and 1 V (right). **b** Variation of the width of the top plateau, observed in the SEM image, as a function of the operating frequency (f_{piezo}) at V_{AC} = 1 V.

In the manuscript, the displacement of the DMB was calculated under a given self-oscillation condition. To verify this, we conducted an additional experiment using a shear mode piezoelectric chip (PL5PB, THORLABS). We placed the DMB on the piezoelectric chip and measured the width of the DMB by applying 1 V to the piezoelectric substrate at different frequencies. Near its resonance condition, the image becomes blurred as observed in Supporting Fig. 10a. And the variation of the width of the top plateau is plotted and fitted by two Lorentzian overlapping functions (Supporting Fig. 10b). They correspond to the resonant frequency of the piezoelectric chip at 1.79 MHz and that of the DMB at 14.27 MHz. It was observed that the displacement of the DMB was 3.7 nm. Using the finite element simulation (COMSOL), the amplitude of the force exerted to the DMB and spring constant were calculated to be approximately 6.5 nN and 1.69 N/m, respectively. Meanwhile, the spring constant calculated using the harmonic oscillator model was 1.687 N/m, confirming the consistency with the results obtained using the piezoelectric chip.

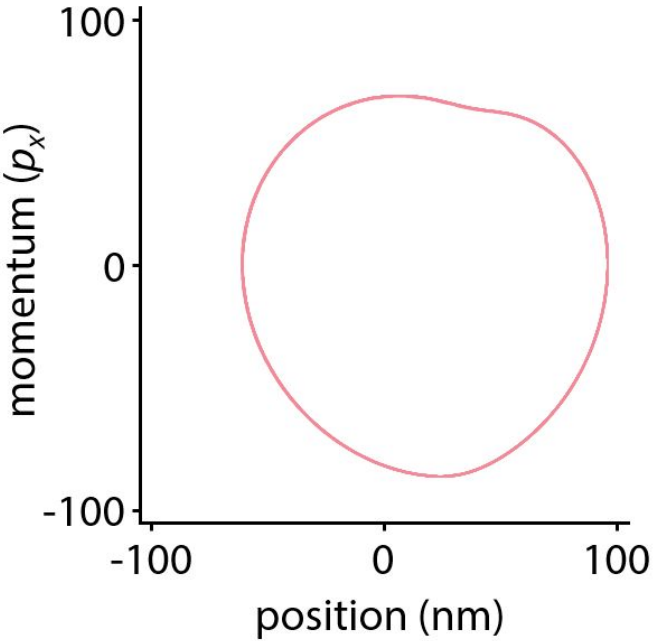

Supporting Figure 11. Phase diagram of the mechanical motion of the DMB, resulting in a limit cycle.

Supporting Information 8: Simulation of tensile strain under the condition of maximum displacement

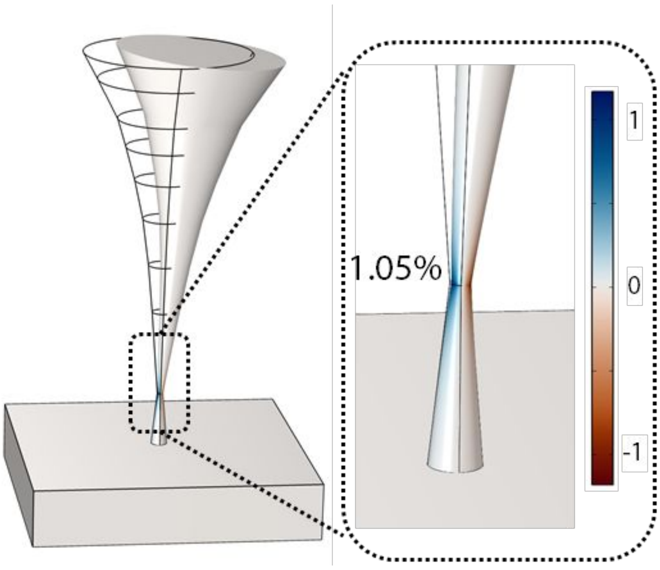

Supporting Figure 12. Finite element analysis of tensile strain in the DMB.

We calculated tensile strain on the DMB during the self-oscillation with the displacement of 100 nm, obtained in Fig. 3b in the main manuscript, using a Finite Element Model (FEM). The tensile strain was found to be 1.05 %. This is significantly lower compared to the reported maximum tensile strain of 13.4 %[6].

Supporting Information 9: Preparation of bacteriophage T4 in solution

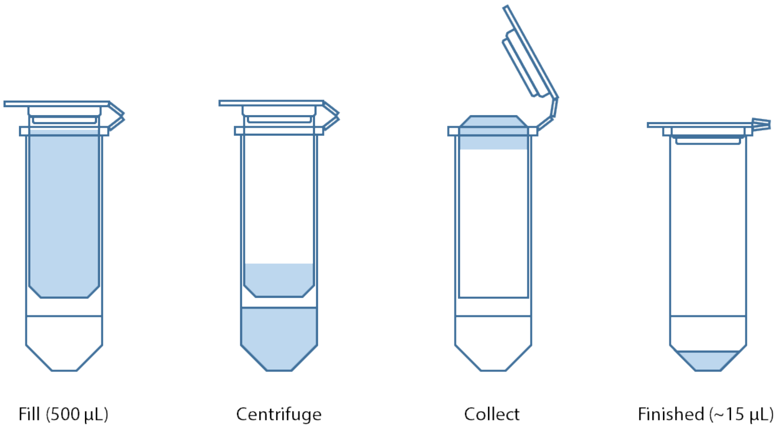

Supporting Figure 13. Concentrating sequence of the bacteriophage T4 in solution.

The bacteriophage T4 was concentrated in solution (Carolina™ Coliphage T4, 5 mL) using a centrifuge (GYLZ-1312, Labogene) and a filter (UFC510024, Millipore). The concentrating sequence is illustrated in Supporting Fig. 13. First, the filter was installed into the tube and the bacteriophage T4 in solution (500 μL) was added. After centrifuging, we re-installed the filter in reverse and centrifuged again to collect the solution retained in the filter. The volume of the solution filtered was about 15 μL, effectively concentrating the solution by 33 times in a single step.

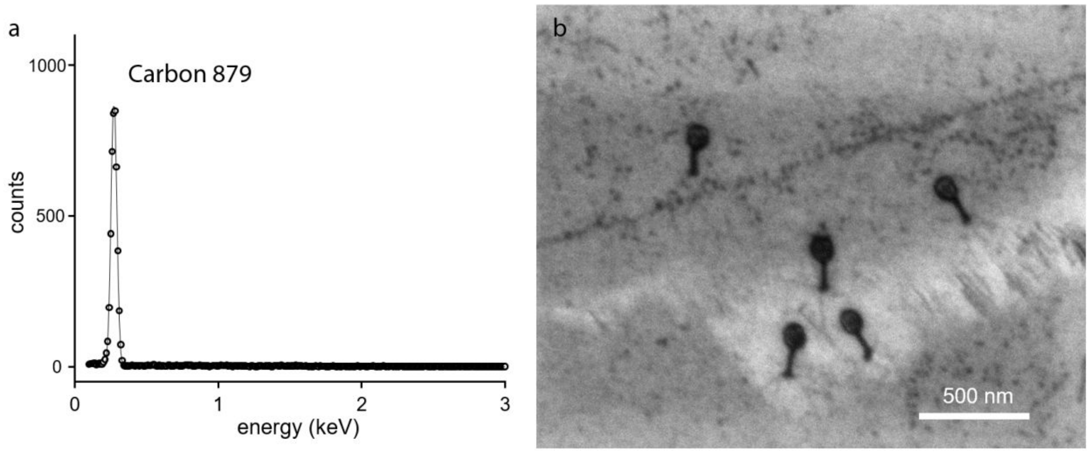

Supporting Figure 14. Energy Dispersive X-ray Spectroscopy (EDS) result. **a** EDS spectrum. **b** A scanning electron micrograph of bacteriophage T4 loaded onto a diamond substrate. After dropping the

solution with bacteriophage T4 virions onto a diamond substrate, the solution was naturally evaporated at room temperature within 30 minutes. The substrate was rinsed with DI water for 5 minutes.

Supporting Information 10: Frequency compensation based on the analyte's position on the top plateau of the DMB

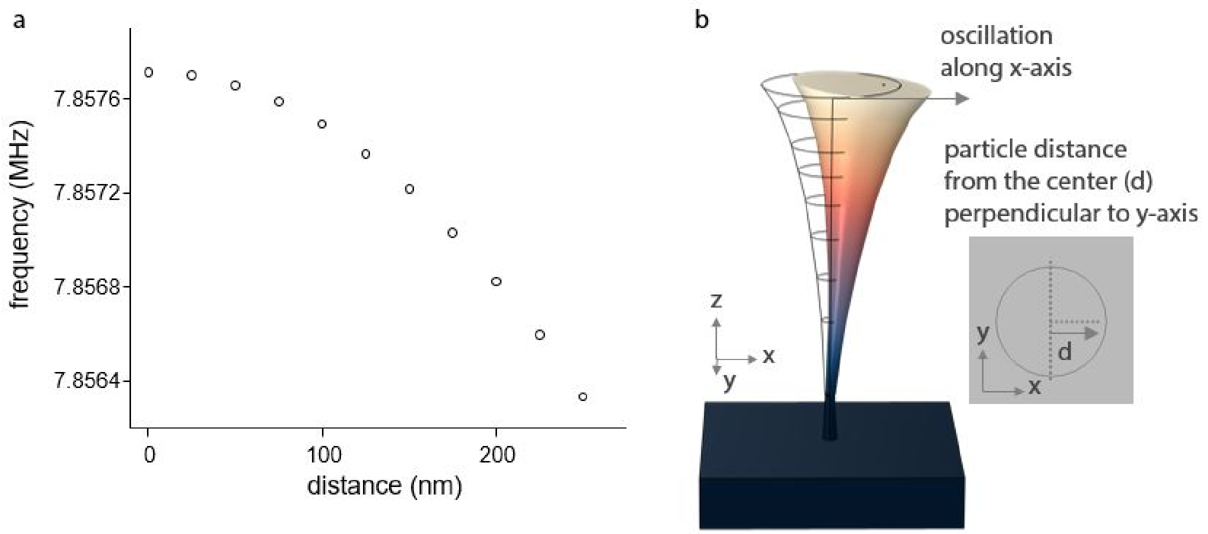

Supporting Figure 15. Frequency compensation depending on the landing position of the analyte. **a** The resonance frequency calculated as a function of the distance from the axis perpendicular to the oscillating direction with the effective mass of 200 MDa. **b** Finite element simulation of the resonance of the DMB. Inset shows the definition of the distance (*d*).

The resonance frequency shifts depending not only on the analyte mass but also on its radial landing position on the DMB, due to the position-dependent contribution to the effective mass.

$$f + \Delta f = \frac{1}{2\pi}\sqrt{\frac{k}{m_{eff} + \Delta m_{eff}(d)}}, \qquad (28)$$

where m_{eff} is the effective mass of the DMB, and $\Delta m_{eff}(d)$ is the mass contribution from the analyte, which depends on its radial position d.

In our system, we extract the landing position of each analyte from SEM imaging and apply a correction based on a pre-characterized function $f(d)$, which was derived through finite element simulations and agrees with the observed experimental trend shown in Supporting Figure 15a. To compensate for this effect, we use the following expression:

$$\Delta m_{eff}(d) = \Delta m_p f(d) \qquad (29)$$

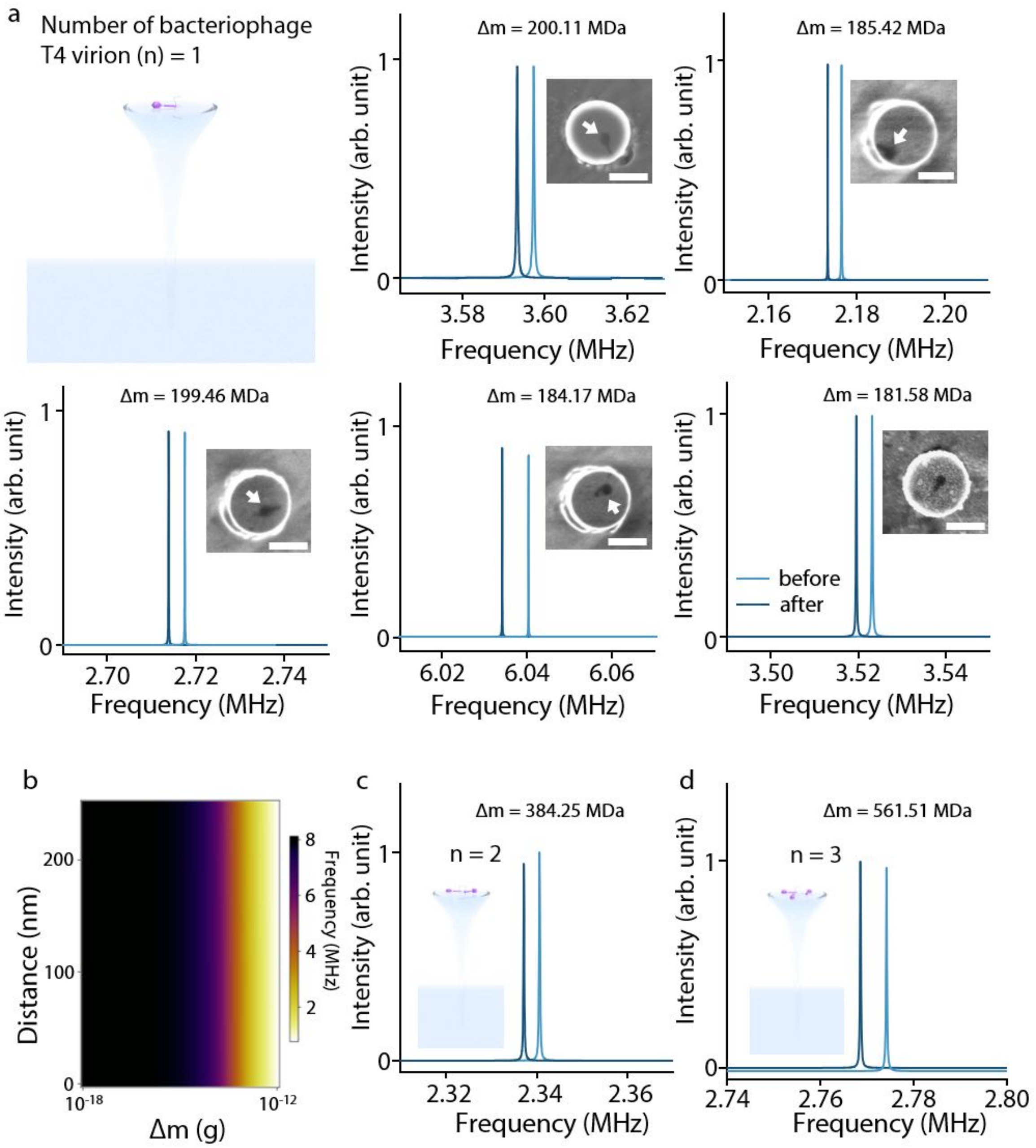

Supporting Figure 16. Mass measurements of individual bacteriophage T4 virions. **a** Illustration of DMB with a single bacteriophage T4 on top and frequency responses with single bacteriophage T4 virions. Insets show the bacteriophage T4 virions on top of the DMBs (Scale bar = 400 nm). **b** Resonance frequency compensation at different masses and distances from the axis perpendicular to the oscillating direction. **c** and **d** Frequency responses with two and three bacteriophage T4 virions.

Supporting Information 11: Mass resolution of the measurement with the DMB

The mass resolution σ of the DMB is determined by the following factors:

1. Frequency stability (σ_A)

To quantify the frequency fluctuation, the Allan deviation of the obtained data was calculated to be $\sigma_A \sim 3 \times 10^{-6}$, corresponding to a change in mass of 0.36 MDa (Fig. 2c in main manuscript).

$$\sigma_A = \delta f / f_0 \quad (30)$$

2. Adsorption-desorption noise (σ_P)

To assess the frequency noise arising from environmental changes during repeated evacuation cycles, we monitored the changes in the measured frequency during successive loading/reloading processes (Supporting Fig. 17). The measurements show a standard deviation of 217.74 Hz, which corresponds to an error range of 3.7 MDa. This noise defines the practical limit of mass resolution in our current system, as it dominates the uncertainty over other sources such as thermal noise or spatial resolution effects.

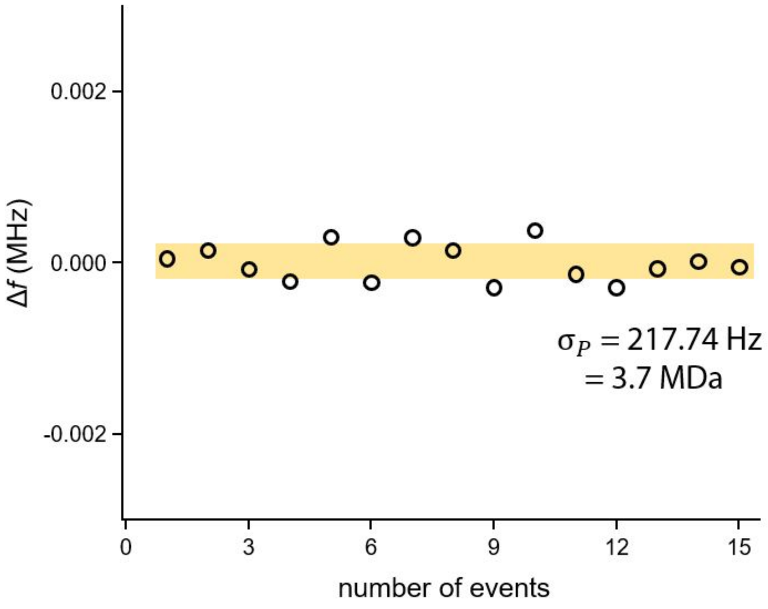

Supporting Figure 17. Frequency changes due to adsorption-desorption noise.

3. Stiffness of the analyte (σ_{sp})

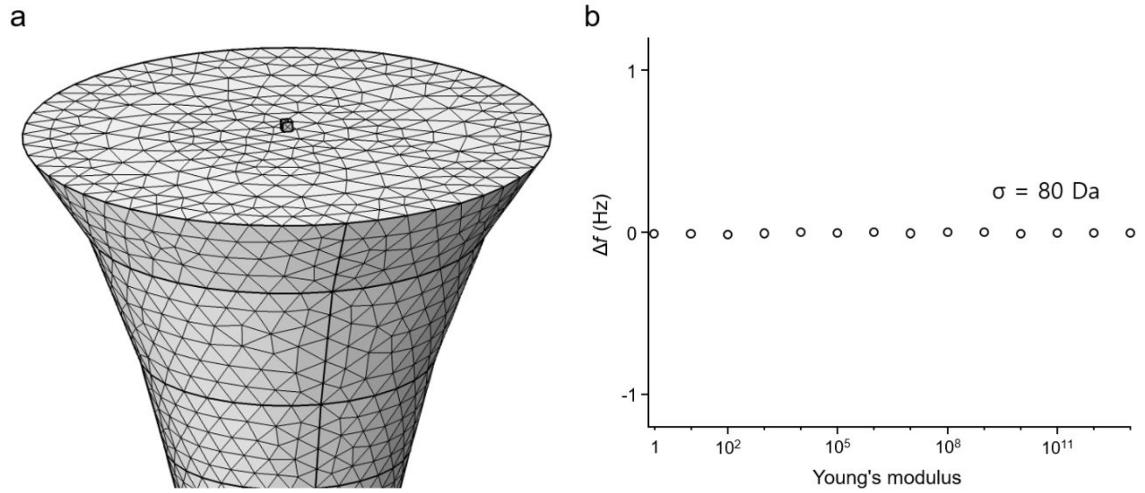

Supporting Figure 18. The stiffness dependence of the DMB's frequency responses. **a** Finite element modeling (FEM) of the DMB with an analyte on top. **b** Frequency responses according to different Young's modulus.

The stiffness of analytes must be considered for an accurate assessment in mass measurement[7]. For this, we placed a particle with a mass of 194 MDa (equivalent to that of the bacteriophage T4) at the center of the DMB's top surface. Then, by varying the Young's modulus of this analyte from 1 Pa to 10 GPa, we calculated the corresponding resonance frequencies. The result, as seen in the Supporting Fig. 18, demonstrates a standard deviation of 0.008 Hz, corresponding to an uncertainty of 80 Da. This indicates that the impact of the analyte stiffness on the frequency measurement of the DMB is negligibly small.

4. Spatial resolution of the scanning electron microscope (σ_{sr})

The spatial resolution of a scanning electron microscope determines the uncertainty in the positional information of the analyte on the DMB. Our SEM has the capability of achieving a spatial resolution of 2 to 10 nm, depending on the measurement conditions. Assuming a spatial resolution of 10 nm with an analyte of 194 MDa, the measurement uncertainty is calculated to be 0.59 Hz, corresponding to 0.015 MDa at the center of the DMB. At edge position (250 nm from the center), this positional uncertainty

translates to a larger mass error of approximately 0.55 MDa. These results indicate the impact of the uncertainty of the positional information from a conventional SEM is negligibly small.

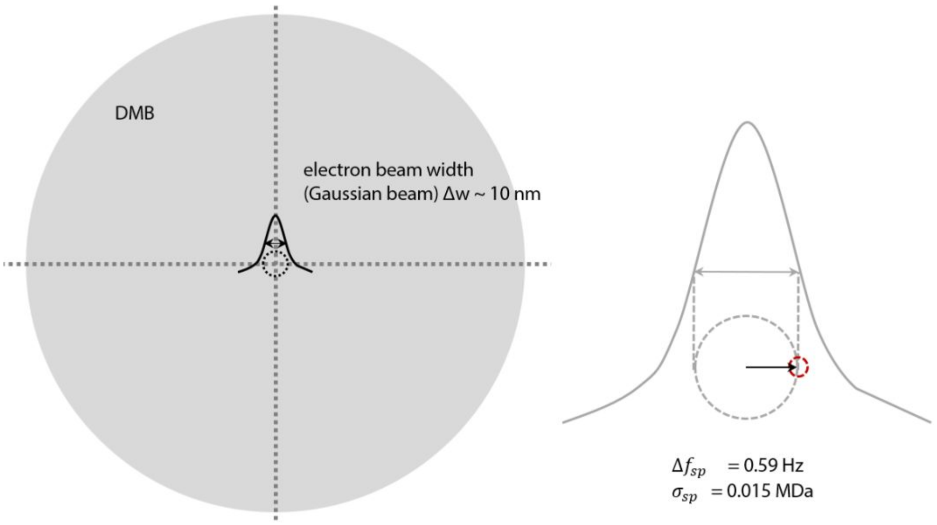

Supporting Figure 19. Spatial resolution of the SEM. This result demonstrates that the spatial resolution of the SEM contributes only minimally to overall mass error.

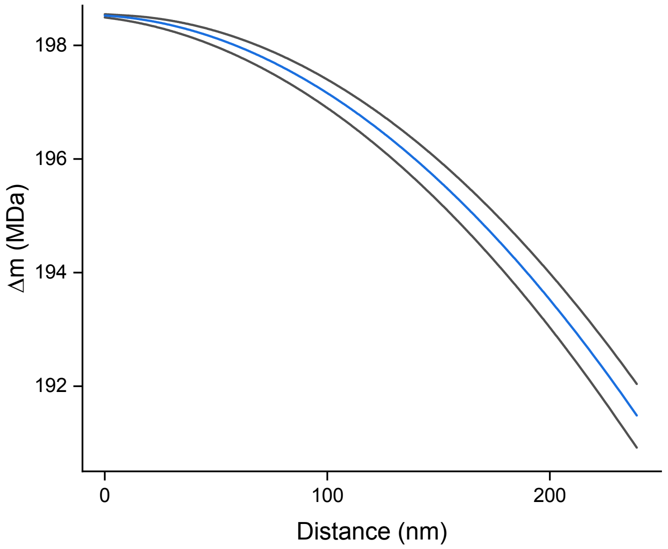

Supporting Figure 20. FEM result of mass error due to SEM spatial resolution at resonance frequency of 7.88 MHz. When a 200 MDa analyte is positioned near the edge of the DMB (250 nm offset), a spatial uncertainty of 10 nm leads to a mass error of 0.55 MDa.

Supporting Information 12: Mass-loading position dependence of frequency response

As the frequency response of the DMB is influenced not only by the mass of the analyte but also by its position on top of the plateau. We deposited carbon composites at two different positions on the plateau of the DMB and monitored the corresponding frequency shifts for each loading. Supporting Figure 20 shows a 0.4 kHz shift for the analyte with the same mass of 150 MDa. As shown in Supporting Fig. 6, the spring constant of the DMB remains almost constant after several analyte deposition processes. This suggests consistency with the calculated position-dependent frequency response (Supporting Fig. 16b).

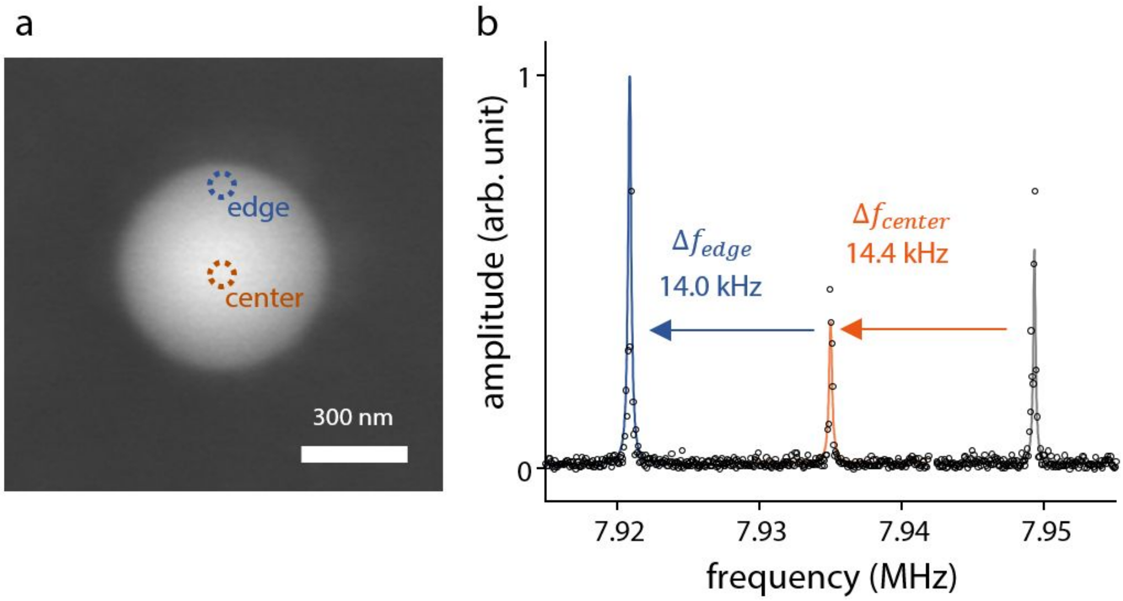

Supporting Figure 21. Frequency responses to the deposition of carbon nanocomposites at the center and edge of the DMB. **a** SEM image of the top plateau of the DMB highlighting the deposition areas. **b** Corresponding frequency shifts for mass loadings at different positions.

Supporting Information 13: Cross section of the DMB array.

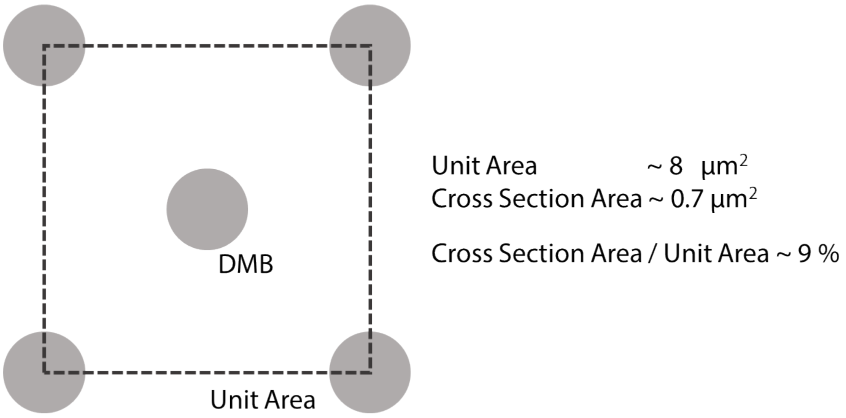

Supporting Figure 22. Cross section of the DMB array. The DMB operates without the need for sizable electrodes for actuation or sensing, thereby enabling a significantly high integration density. The area of the top plateaus in the DMB array is calculated to be 0.7 μm^2. This implies that approximately 9 % of analytes from a solution drop-cast are available for mass evaluation.

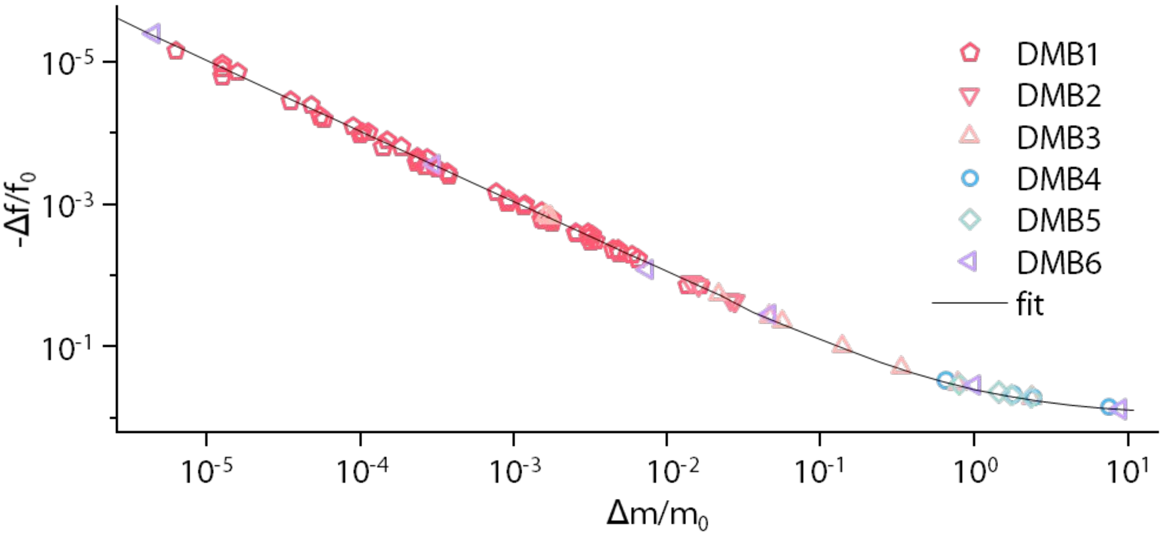

Supporting Figure 23. Log–log plot of resonance frequency detuning versus loaded analyte mass. The graph presents the same data as Figure 3a in the main manuscript, displayed on a log–log scale to enhance the visibility of both low- and high-mass regimes.

Supporting References:

1 D. Gabor. Theory of communication. Part 3: Frequency compression and expansion. *J. Inst. Electr. Eng.*, **93** (1946).

2 Frigo, M. & Johnson, S. G. The design and implementation of FFTW3. *Proc. IEEE* **93**, 216–231 (2005).

3 Riley, W. J. (2008). Handbook of Frequency Stability Analysis. *NIST Special Publication* 1065. Available at: https://tf.nist.gov/general/pdf/2220.pdf

4 Mulders, J. J. L., Belova, L. M. & Riazanova, A. Electron beam induced deposition at elevated temperatures: compositional changes and purity improvement. *Nanotechnology* **22** (2011).

5 Demers, H. *et al.* Three-Dimensional Electron Microscopy Simulation with the CASINO Monte Carlo Software. *Scanning* **33**, 135-146 (2011).

6 Nie, A. M. *et al.* Approaching diamond's theoretical elasticity and strength limits. *Nat Commun* **10** (2019).

7 Dominguez-Medina, S. *et al.* Neutral mass spectrometry of virus capsids above 100 megadaltons with nanomechanical resonators. *Science* **362**, 918-922 (2018).
